# Can the Pfannenstiel skin incision length be adjusted according to the fetal head during elective cesarean delivery?

**DOI:** 10.3389/fsurg.2023.1227338

**Published:** 2023-09-26

**Authors:** Baris Kaya, Ozlen Emekci Ozay, Ali Cenk Ozay, Abdullah Tüten

**Affiliations:** ^1^ Department of Obstetrics and Gynecology, Basaksehir Cam and Sakura State Hospital, Istanbul, Türkiye; ^2^ Department of Obstetrics and Gynecology, Cyprus International University School of Medicine, Lefkosa-TRNC, Mersin, Türkiye; ^3^ Department of Obstetrics and Gynecology, Cerrahpasa University School of Medicine Hospital, Istanbul, Türkiye

**Keywords:** cesarean delivery, Pfannenstiel incision, incision length, occipitofrontal diameter, Aesthetic cesarean incision

## Abstract

**Objective:**

The study aims to determine whether the Pfannenstiel skin incision can be adjusted according to the fetal head's occipitofrontal diameter (OFD) during primary cesarean delivery.

**Background:**

Eligible 114 nulliparous women delivered at term by cesarean section in which Pfannenstiel skin incision was performed according to the OFD of the fetal head between June 2017 and September 2021 were included. Excluded cases were non-vertex presentations, all emergency cesarean sections, severe preeclampsia, women in an active phase of the first stage of labor and second stage of labor, placenta previa and low-lying placenta, multiple pregnancies, and uncontrolled gestational diabetes mellitus.

**Results:**

Among 114 eligible nulliparous women, the mean OFD was 116.1 ± 7.2 (99–138) mm, and the measurement of the Pfannenstiel skin incision length, which was performed according to the OFD was found to be 122.8 ± 9.2 (100–155) mm. The difference between OFD and Pfannenstiel incision kept remained within 10 mm in 90 (82.5.2%), 10–20 mm in 17 (15.5%), and more than 20 mm in two women (1.8%). This technique was successful in 109 (95.6%) out of 114 women without extending the skin incision. In five women, skin incision needed to be extended up to 38 mm. In 10 women (8.7%), the rectus abdominis muscle was cut partially to deliver the fetal head. The mean fetal umbilical artery pH was 7.33 ± 0.05. No neonatal hypoxia was encountered in the study.

**Conclusion:**

Pfannenstiel skin incision can be adjusted according to the OFD with minimal margins of error. This technique may provide better cosmetic results by avoiding unnecessarily prolonged incisions with similar newborn outcomes.

**Clinical Trial Registration:**

Clinicaltrials.gov, identifier [NCT05632796].

## Introduction

Cesarean birth is the extraction of a baby by cutting off the abdominal wall layers to the uterus, dating back to ancient BC. Cesarean surgical techniques only evolved a little since the foremost surgical procedures were introduced as uterine lower segment transverse incision (1, 2), namely, Kerr incision (3), and the lower abdominal transverse skin incision, namely, Pfannenstiel incision (4). Pfannenstiel incision is the most frequent abdominal incision used for cesarean delivery (CD) because of its cosmetic advantages due to the level of incision (bikini cut) and the use of Langer's lines (5). Surprisingly, Pfannenstiel incision’s length has been out of interest since its introduction in 1900. Its length should be enough for the surgeon to feel comfortable and provide enough exposure during the operation (6). In the past, although a 15 cm Pfannenstiel incision length was claimed to be enough (7, 8), the given Pfannenstiel incision length ranged widely between 11.7 and 16.9 cm (7, 9–13). More than 20 cm Pfannenstiel incision length has also been reported (14).

Nevertheless, some obstetricians tend to make the incision as short as possible in the contemporary world, considering the patient's esthetic expectations. Shorter incisions may not have cosmetic advantages only; pain, wound infection, and nerve entrapment may also be less (10–12, 15–17). In addition, abdominal laparotomy incisions should be kept as small as possible to reduce surgical stress (18). To date, the length of the Pfannenstiel incision remains to be determined.

This study aims to describe a modified Pfannenstiel skin incision technique that was adjusted according to the fetal head's occipitofrontal diameter (OFD) and evaluate its short-term maternal and neonatal outcomes.

## Materials and methods

Nulliparous women who underwent primary CD in which the Pfannenstiel incisions were performed according to the occipitofrontal diameter between June 2017 and September 2021 at Yakın Dogu University Hospital were evaluated. This interventional pilot study was approved by the Institution's Local Ethics Committee (30.09.2021, Number: YDU 2021/95-1398) and was registered to clinicaltrials.gov on 01/12/2022 with the registry number (NCT05632796).

The information regarding the procedure was given, and informed consent was obtained from all women.

The women older than 18 years with term nulliparous pregnancies (>37 weeks) who underwent primary CD with vertex presentation under spinal anesthesia were included.

During the evaluation of medical records, all emergency cesarean sections, including women with non-reassuring fetal heart rates and fetal distress, third trimester bleeding including abruption of placenta and placenta previa bleeding, arrest of labor in the second phase, and severe preeclampsia, were excluded. In addition, women who underwent CD with non-vertex presentations such as breech and shoulder presentation, deflexion fetal head presentations such as face and brow, those who were in the active phase of the first stage of labor, who had placenta previa and low-lying placenta, multiple pregnancies, and uncontrolled gestational diabetes mellitus were also excluded.

Primary outcomes were the final incision length following the Pfannenstiel incision skin closure, the need to extend the skin incision by the surgeon, and the difference between the initial length of the Pfannenstiel incision, which was made according to the fetal OFD and the final one. Secondary outcomes were surgical features, including duration of operation, intraoperative difficulties or additional surgical interventions during delivery of the baby, neonatal outcomes, and postoperative maternal outcomes, including wound complications.

## Surgical technique

Adjusting the Pfannenstiel skin incision according to the fetal OFD technique was invented and first performed by the first author (BK); it was introduced to authors OO and AO. In this study, the three surgeons performed cesarean deliveries (BK, OO, and AO).

**Figure 1:**
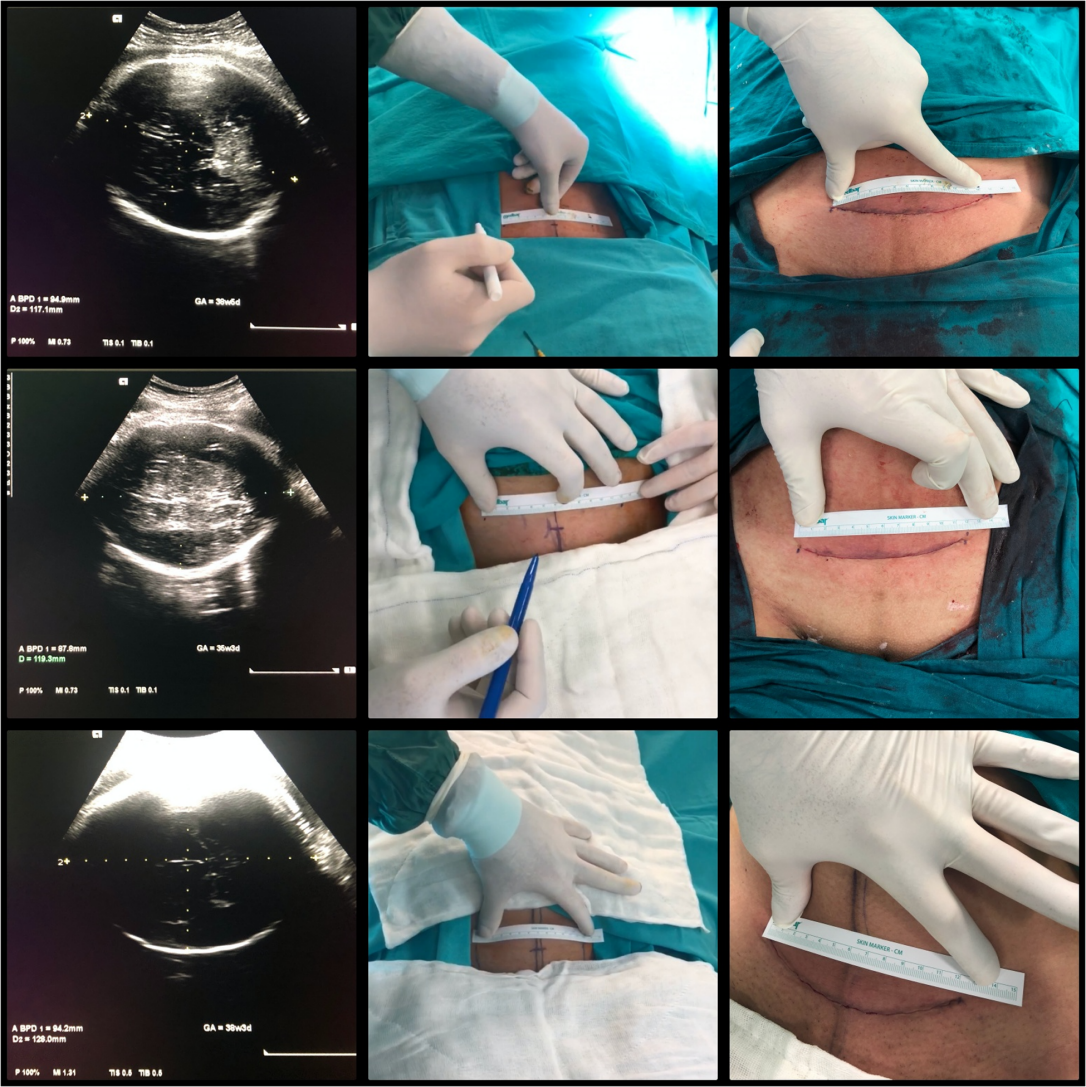
Adjusting the Pfannenstiel skin incision according to the ultrasound measurement of occiputofrontal diameter (OFD). (Pfannenstiel incision with Kaya modification). OFD's were measured as 117, 119 and 128 mm from top to bottom, respectively. Pfannenstiel incisions after closure of the skin were measured as 128, 117 and 125 mm from top to bottom, respectively.

All women underwent ultrasound measurement of OFD and other fetal biometric measurements, including biparietal diameter (BPD), head circumflex (HC), and estimated fetal weight (EFW), just before the operation. According to the weight of pregnant women, 1–3 g of first-generation cephalosporin was given within 1 h of the skin incision. All women were subjected to regional spinal anesthesia. The abdominal skin was cleaned with a povidone-iodine solution, and the operative field was covered with sterile surgical drapes. Initially, the skin 2–3 cm above the pubic bone, where the Pfannenstiel incision will be made, was marked with the sterile skin marker pen according to the OFD measurement. The line was measured with a flexible ruler (Medbar® skin marker kit, Izmir, Türkiye). A slightly cephalad curved Pfannenstiel incision was made according to the OFD, marked on the skin (Pfannenstiel incision with Kaya modification). The fascia incision was made several centimeters more expansive than the marked skin incision (not measured). Following direct entry to the abdominal cavity with the right index finger, care was taken not to stretch the skin to avoid extending the skin incision length. The uterine lower segment Kerr incision was performed with a scalpel and opened enough with two thumbs of the surgeon to allow fetal head delivery. Fundal pressure with appropriate strength was applied to deliver the baby. Following the baby's delivery, the umbilical cord was cut, and an arterial umbilical cord blood sample was taken and sent for umbilical artery pH measurement. The pediatricians evaluated the 1- and 5-min Apgar scores in the operation room. Placentas were delivered either by the extra-abdominal placental delivery method (19) or spontaneous intraabdominal delivery up to the surgeon's preference. Uterine incision was double-layer sutured with 1 polyglactin 910 suture (Vicryl®, Ethicon, Somerville, NJ, USA). Intravenous infusion of 20 IU oxytocin in 500 ml of normal saline at a rate of 125 ml/h was started during the cesarean section and continued for 4 h after delivery. Peritoneum and fascia were closed with 2-0 and 1 polyglactin 910 suture (Vicryl®, Ethicon, Somerville, NJ, USA), respectively. The skin incision was sutured with continuous 3-0 rapid polyglactin 910 suture (Vicryl®, Ethicon, NJ, USA) and measured with a flexible ruler (Medbar® skin marker kit, Izmir, Turkey) in centimeters following completing the skin incision closure (Figure 1) (see Supplementary Videos S1, S2). The duration of the operation was accepted as the time between the incision and the skin closure. The time interval between the uterine incision and the baby's delivery was also recorded. The wounds were kept dressed until 24 h and Transparent Film spray was applied (Opsite® spray, Smith & Nephew, Belgium) following cleaning with iodine solution and then left open. Wounds were checked 1 and 6 weeks after cesarean delivery.

### Statistical analysis

The Statistical Package for the Social Sciences (SPSS) software version 22.0 (SPSS Inc., Chicago, IL, USA) was used for establishing the database and statistical calculations. The Kolmogorov–Smirnov test was used to evaluate the homogeneity of the distribution of continuous variables. Homogenous continuous variables were given as mean ± standard deviation (SD), calculated with one-sample *t*-test in all cases, and compared in subgroups with independent samples *t*-test. Heterogenous continuous variables were given as median (minimum–maximum), calculated with the one-sample Kolmogorov–Smirnov test in all cases, and compared in subgroups with the Mann–Whitney *U*-test. The categorical variables were given as n/N (%) calculated with descriptive analysis. Pearson correlation analysis was performed for the parameters affecting the Pfannenstiel incision–OFD difference. A *p*-value of <0.05 was accepted as statistically significant.

## Results

During the study period, a total of 556 cesarean deliveries were performed by the three surgeons (BK, AO, and OO). About 190 previous cesarean deliveries were excluded, and 336 women underwent primary cesarean delivery. Among the 336 primary cesarean deliveries, 210 women were excluded due to arrest of labor, fetal distress, malpresentation, multiple pregnancies, and third trimester bleeding. Finally, 115 cesarean deliveries were performed with the modified Pfannenstiel skin incision out of 156 eligible primary cesarean deliveries. In one patient, the skin incision needed to be extended following the delivery of the baby due to Bakri balloon implementation for uterine atony.

**Figure 2:**
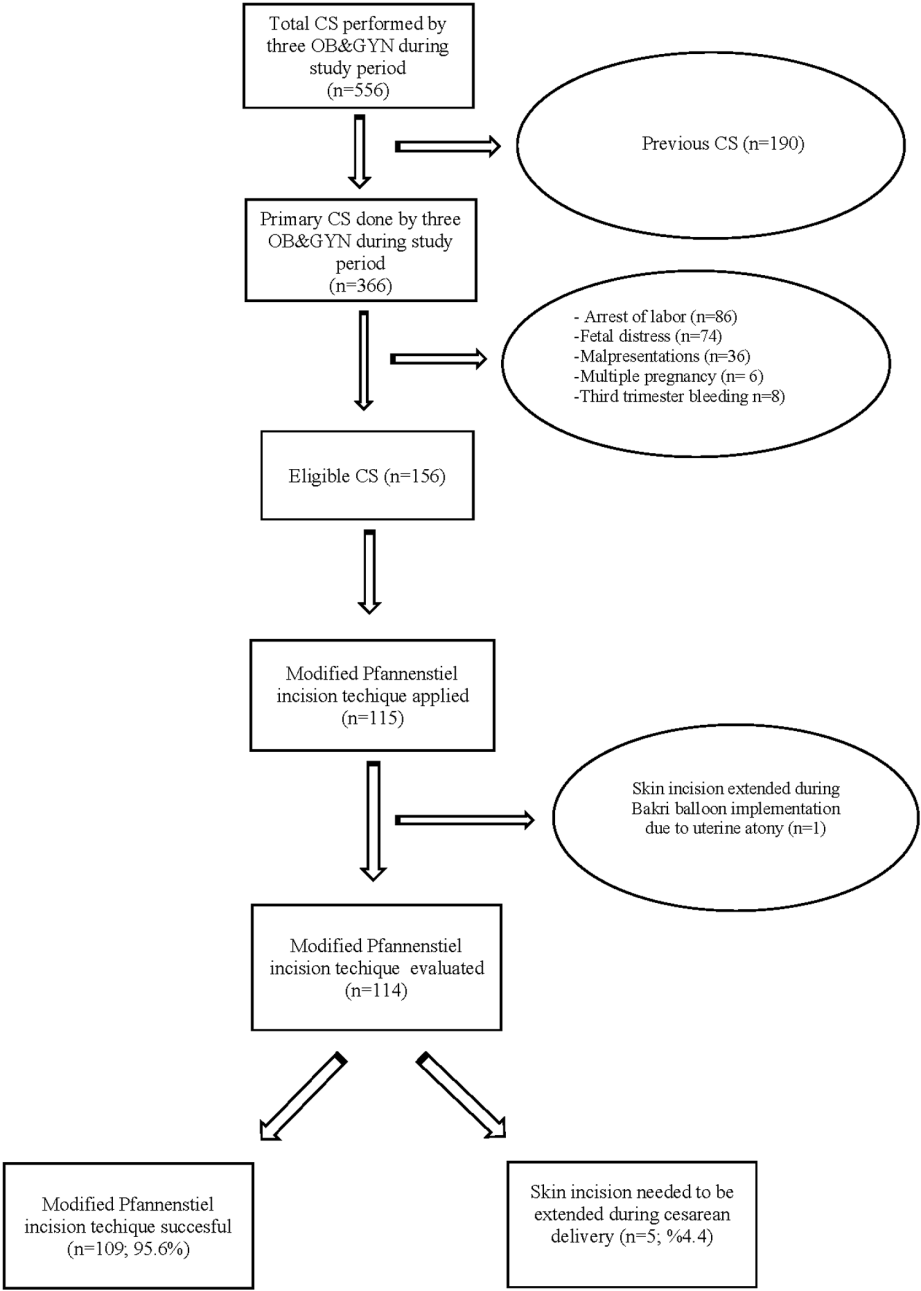
Flowchart of the study.

This technique was completed in 109/114 (95.6%) women without extending the skin incision (Figure 2). In five women (4.4%), Pfannenstiel skin incision needed to be extended during cesarean delivery, which was accepted as a technique failure (Table 1). Demographic characteristics of women, fetal ultrasonographic measurements, operation features, preoperative and postoperative hemoglobin levels, and neonatal outcomes of the study population are given in Table 2. The indications of CD and diseases accompanying pregnancy are given in Table 3. The Pfannenstiel skin incision, made according to the fetal OFD, was approximately 116.1 ± 7.2 mm (99–138 mm), and the final measured Pfannenstiel skin incision length after closing the skin was found to be 122.8 ± 9.2 mm (100–155 mm). The mean difference between the final and initial incisions was 6.8 ± 5.6 mm.

**Table 1:** Outcomes of the study.

Total number	114
Success	109 (95.6%)
Partial rectus cut^a^	7 (6.4%)
Bilateral	3
Unilateral	4
Failure	5 (4.4%)
Skin incision extension alone	2
Skin incision extension + partial rectus cut^b^	3

^a^ Rectus abdominis was cut partially (unilateral or bilateral) to deliver the fetal head successfully without skin incision extension.

^b^ In one case, rectus abdominis was cut bilaterally and additionally epigastric inferior artery was ligated.

**Table 2:** Maternal, fetal and perioperative features of the study population.

	*N*	Mean ± standard deviation	Median (minimum–maximum)
Maternal age (years)	114	28.9 ± 4.7	29 (20–41)
BMI (kg/m^2^)	114	28.7 ± 3.3	28.2 (23.1–42.2)
Gestational age (weeks)	114	38.5 ± 0.8	38.7 (37–40.8)
EFW (g)	114	3,308 ± 377	3,285 (2,450–4,200)
BPD (mm)	114	93.1 ± 3.2	93.7 (86.6–101.3)
OFD (mm)	114	116.1 ± 7.2	116.5 (99–138)
HC (mm^3^)	114	335.9 ± 14.2	336.3 (298–376.3)
AC (mm^3^)	114	335.4 ± 17.0	333.5 (284.0–373.0)
Pfannenstiel incision (mm)	114	122.8 ± 9.2	122 (100–155)
Pf-OFD (mm)	114	6.8 ± 5.6	6 (−3–28)
UI-FD interval (s)	114	52.5 ± 32.9	40 (20–200)
Duration of operation(min)	114	44.1 ± 12.1	42 (30–116)
Fetal weight (g)	114	3,289 ± 359	3,275 (2,400–4,045)
Apgar 1st min	114	8.4 ± 0.7	9 (7–9)
Apgar 5th min	114	9.7 ± 0.5	10 (8–10)
Umbilical artery pH	114	7.33 ± 0.04	7.33 (7.17–7.49)
Pre-operation Hb (g/dl)	114	11.6 ± 1.1	11.8 (8.9–14.4)
Post-operation Hb (g/dl)	114	10.6 ± 1.0	10.5 (8.0–14.1)
Hospital stay (day)	114	1.93 ± 0.28	2 (1–4)

Pf, Pfannenstiel; UI, uterine incision; FD, fetal delivery; Hb, Hemoglobin.

All values are presented as mean ± standard deviation and median (minimum–maximum).

**Table 3:** Cesarean indications and diseases accompanying pregnancy among 114 women.

Cesarean indications	Number of women (%)
Maternal request total	75 (65.2)
Before onset of labor	60 (52.1)
After onset of labor	7 (6.0)
IVF	4 (3.4)
Advanced maternal age (35>)	4 (3.4)
Suspicious of macrosomia	7 (6.0)
Genital condyloma	2 (1.7)
Suspicious of CPD	30 (26.1)
Diseases accompanying pregnancy^a^	Number of women *n* (%)
GDM receiving insulin	3 (2.6)
Gestational hypertension	1 (0.8)
Hypothyroidism	4 (3.4)
Epilepsy	1 (0.8)
Cardiac arrhythmia	1 (0.8)
Gilbert syndrome	1 (0.8)
Asthma	1 (0.8)
Gallbladder stone	1 (0.8)

IVF, *in vitro* fertilization; CPD, cephalopelvic disproportion; GDM, gestational diabetes mellitus.

^a^ In some women, more than one disease was seen concomitantly.

**Figure 3:**
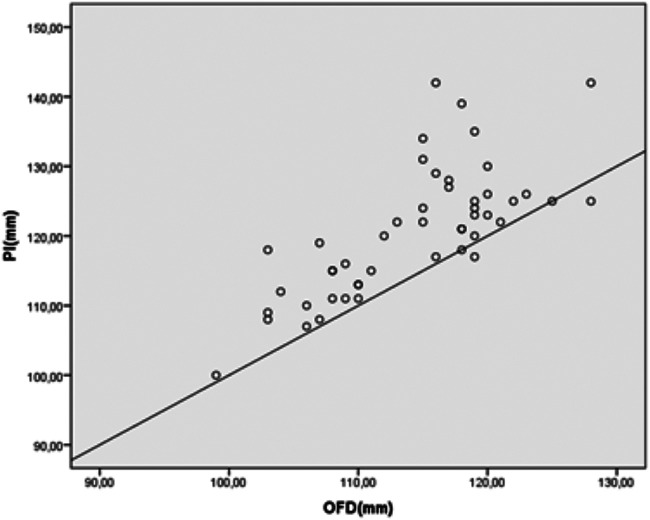
Pfannensitel incision and OFD values of the study population shown in a Scatter Plot Graphic. PI, pfannenstiel incision; OFD, occipitofrontal diameter.

Figure 3 shows the relationship between the OFD and Pfannenstiel incision range. The difference between the OFD and the final Pfannenstiel incision kept remained within 10 mm in 90 (82.5%), 10–20 mm in 17 (15.5%), and more than 20 mm in two women (1.8%).

In the study, any correlation was not found between the Pfannenstiel incision length and features, including maternal age, body mass index (BMI), fetal weight, and fetal ultrasonographic measurements (Table 4).

**Table 4:** The correlation analysis of Pfannenstiel incision–OFD difference according to the demographic, clinic, and ultrasound parameters.

	*r*	*p*
Maternal age	−0.003	0.972
BMI	0.009	0.925
Gestational age	0.088	0.361
EFW	0.101	0.296
BPD	0.181	0.058
HC	0.021	0.824
AC	0.000	0.997
Birth weight	0.042	0.666

*p* < 0.05 is considered statistically significant.

There was no statistical difference in terms of fetal ultrasonographic measurements, including OFD, BPD, HC, as well as fetal weight, neonatal outcomes, and also maternal features between the ≤1 cm group (*n* = 90) and >1 cm group (*n* = 19) (Table 5).

**Table 5:** Comparison of maternal, fetal, and perioperative features of groups between the initial and final Pf incision ≤1 and >1 cm.

	Initial–final Pf incision ≤1 cm (*n*:90)	Initial–final Pf incision >1 cm (*n*:19)	*p*
Maternal age (year)	28.8 ± 4.6	29.6 ± 5.5	0.484
BMI (kg/m^2)^	28.8 ± 3.4	28.1 ± 3.1	0.665
Gestational age (week)	38.5 ± 0.8	38.7 ± 0.8	0.334
EFW (g)	3,268 ± 354	3,367 ± 404	0.283
BPD (mm)	92.6 ± 4.1	93.7 ± 2.6	0.252
OFD (mm)	113.0 ± 7.0	113.3 ± 6.9	0.091
HC (mm^3^)	335.7 ± 13.8	333.1 ± 14.1	0.459
AC (mm^3^)	334.7 ± 15.9	333.1 ± 19.9	0.707
Pf incision (mm)	120.7 ± 7.7	128.3 ± 8.8	0.001*
PI-OFD (mm)	4 (−3–10)	13 (11–26)	0.001*
UI-FD interval (s)	36 (20–200)	49 (20–130)	0.529
Duration of operation (min)	42 (30–94)	40 (30–50)	0.119
Fetal weight (g)	3,280 ± 339	3,209 ± 410	0.425
Apgar 1st min	9 (7–9)	9 (7–9)	0.306
Apgar 5th min	10 (9–10)	10 (9–10)	0.106
Umbilical artery pH	7.34 ± 0.04	7.32 ± 0.03	0.075
Preoperative Hb (g/dl)	11.6 ± 1.0	12.0 ± 1.2	0.179
Postoperative Hb (g/dl)	10.5 ± 1.1	10.7 ± 1.2	0.581
Hospital stay (day)	2 (1–4)	2 (1–2)	0.991

Values are presented as means ± standard deviation or median (minimum–maximum).

Two women with rectus incision and skin incision were excluded from the comparison and discussed separately in the text.

* *p *< 0.05 is considered statistically significant.

In five cases (4.4%), the Pfannenstiel incision arranged according to the fetal OFD had to be extended with scissors between 10 and 38 mm and accepted as a technique failure. In three of these five women, the rectus abdominis muscle was partially cut to deliver the fetal head before Pfannenstiel skin incision extension. In two women, only skin incisions were extended to deliver the baby.

In the failed cases (*n* = 5), the mean fetal measurements, including fetal weight, were higher than the mean measurements of successful cases (*n* = 109) [BPD (96.7 ± 3.7 vs. 92.8 ± 3.9; *p* = 0.030), OFD (115.8 ± 7.1 vs. 122.85 ± 8.9; *p* = 0.042), HC (335 ± 13 vs. 349 ± 17; *p* = 0.032), abdominal circumflex (AC) (334 ± 16 vs. 357 ± 12; *p* = 0.003), fetal weight 3,265 (2,400–4,065) vs. 3,660 (3,600–4,200); *p* = 0.003] (Table 6).

**Table 6:** Maternal, fetal, and perioperative features of the failed cases.

Case no	Age	BMI (kg/m^2^)	Gest. week	C/S indication	Initial–final incision (mm)	Additional features	Apgar score 1–5 min	Umbilical pH	BPD (mm)	HC (mm)	AC (mm)	Fetal weight (gr)
Case1	31	29.4	39 + 0	CPD	118–139	Bilat. Pars. Rectus Abd. cut + A. Epigastrica Inf. Lig.	7–9	7.29	101	354	356	3,600
Case 2^a^	23	30.1	38 + 0	CPD	128–142	Unilat. Pars. Rectus Abd. cut	7–9	7.31	100	369	370	3,640
Case 3	32	26.8	38 + 2	Maternal request	127–165		7–10	7.33	93.5	347	336	3,660
Case 4	25	29.5	40 + 0	Slow progress + macrosomia	131–140		9–10	7.40	93.5	355	358	4,020
Case 5^b^	25	26.6	40 + 3	CPD	109–119	Unilat. Pars. Rectus Abd. cut	7–9	7.32	95.1	321	364	3,850

C/S, cesarean section.

^a^ Neonate stayed at NICU for 1 day and received nasal oxygen due to transient tachypnea of the newborn.

^b^ Uterine atony responded to uterotonic agents.

In seven women, the rectus abdominis muscle was partially cut (unilaterally or bilaterally) without the need for an extension of the Pfannenstiel skin incision. There was no epigastric inferior artery ligation performed. When we look at the rectus-cut and without-cut women, maternal and fetal features, including fetal head diameters and fetal weight, were similar in both groups. Due to difficulty during cesarean delivery of the fetus in this seven women, uterine incision to fetal delivery time was longer [36 s (20–130) vs. 128 s (28–195); *p* = 0,001]. Despite 5-min Apgar scores and umbilical pH levels of the newborns being statistically significantly lower than the women without rectus-cut cases [5-min Apgar scores 10 (8–10) vs. 9 (9–10), *p* = 0.013; umbilical pH: 7.34 ± 0.03 vs. 7.28 ± 0.07; *p* = 0.001], neither fetal hypoxia nor fetal acidosis was encountered in the cases.

Three postpartum hemorrhages due to uterine atony were encountered among the study population. One was excluded from the study because of the skin incision extension during the Bakri balloon implementation due to uterine atony. In the other one, the B-Lynch compression suture was performed without needing to extend the skin incision and therefore was included in the study. The last one responded to uterotonic treatment. Blood and blood products were transfused in both. Neither endometritis nor wound infection was encountered in this study.

No neonatal asphyxia was encountered among the newborns. Five babies were followed up at the neonatal intensive care unit (NICU). One was diagnosed with Hirschsprung disease and kept in NICU for 20 days. One neonate was admitted to the NICU due to hypoglycemia and stayed for 3 days. Three newborns were admitted to the NICU and received treatment with nasal oxygen ± continuous positive airway pressure (nCPAP) due to transient tachypnea of the newborn (TTN), with a stay duration ranging from 1 to 6 days. No endotracheal intubation was needed for any newborn.

## Discussion

In this study, in 109 out of 114 women, cesarean delivery was successfully (95.6%) performed with minimal margins of error via the obtained Pfannenstiel skin incision length according to the ultrasonographic measurement of the OFD of the fetal head. In 90 out of 109 women (82.5%), the difference between the skin incision performed according to the OFD and the final Pfannenstiel incision was maintained within 1 cm.

Our study results showed that if the Pfannenstiel incision is adjusted according to the fetal OFD, the incision length will be approximately 12 cm. Cahana et al. (12) classified the Pfannenstiel incision lengths of the women with repeated CD as short <14 cm, medium = 14–17 cm, and long >17 cm. In their study, among 545 women, approximately two-thirds of Pfannenstiel incisions were between 14 and 17 cm (12). Recently, a multicenter study from the USA among 1,916 women reported the mean Pfannenstiel incision length as 15.3 ± 2.3 cm. The incision length was less than 14.5 cm in one-third of the women, while in one-quarter of the women, the incision length was measured at more than 16.5 cm (13). Unlike the above studies from the USA, Giacalone et al. (9) measured the Pfannenstiel incision as 11.7 ± 1.4 cm among 54 women in France, which also proves that surgeons from different regions of the world have a distinct tendency for the cesarean incision lengths.

The question is whether a universal incision length in clinical practice can be applied to all women worldwide. Studies showed that European newborns’ fetal heads had larger head circumferences than South Asian and Middle East/North African ethnicities (20). In contrast, Asian newborns were smaller and had lower birthweight than ethnic Europeans (21). Moreover, one's anthropometric features and socioeconomic factors can differ from another even in the same ethnicity, affecting fetal biometric measurements and fetal growth (22–25). It has been shown that fetal head size was influenced by features such as maternal age, height, weight, parity, and fetal gender; however, AC and femur length (FL) were both correlated with maternal weight (25). Therefore, a 15 cm Pfannenstiel incision length, as suggested previously (7, 8), might be too wide for Asian women (or women with similar body types) or any women carrying a baby with average dimensions.

The significant advantage of the current technique is: it can be applied to women of all kinds of body types and ethnicities.

Extending the skin incision during fetal head delivery was an accepted failure of the technique in this study. In five (4.4%) out of 114 cases, the surgeons needed to extend the skin incisions, where rectus abdominis muscle was cut partially in three cases. Reasons for the difficulty of delivering the fetal head can be an unengaged or floating head as well as tight or contracted rectus abdominis (probably regional anesthesia effect), maternal features, or the fetal head size and fetal weight.

The number of failed cases is relatively few in the study; however, we may speculate that in women with high BPD, HC, and AC measurements (97th percentile), OFD cannot accurately estimate the Pfannenstiel skin incision.

Another reason for unsuccessful attempts may be some aggressive efforts during cesarean delivery or any degree of fetal head extension due to maneuvers that can cause difficult delivery and failure of the current technique.

In seven out of 114 women (6.1%), delivery of the fetuses was difficult during the operation; therefore, the rectus abdominis muscle was partially cut (unilateral, *n* = 3; bilateral, *n* = 4) without extension of the skin incision. Tight or too contracted rectus abdominis (especially with regional anesthesia) can cause difficult delivery of the fetus during cesarean delivery. They may require excessive maternal fundal pressure applied by the assistance or, in some cases, the rectus abdominis muscle can be cut to deliver the baby. Nevertheless, in wider Pfannenstiel incisions, despite the rectus abdominis muscle being tight or contracted, the opening of the incision may allow the muscle sheets to be retracted by the fingers of the assistants or instruments. However, in the current technique, since the Pfannenstiel incision was relatively shorter, the rectus abdominis muscle must be partially cut (unilateral or bilateral) to deliver the baby's head. There are no complications due to a partial rectus muscle cut; clinical and isokinetic testing for abdominal wall strength showed that the rectus abdominis muscle cut has no harm to the mother compared with the Pfannenstiel incision alone (9). In our study, the time interval between the uterine incision and delivery of the fetus was prolonged, and 5-min Apgar scores and umbilical pH levels were lower in the rectus-cut cases; however, neither fetal hypoxia nor fetal acidosis was encountered.

Apart from the time-honored Pfannenstiel incision for CD, some modifications were made in terms of the higher level of skin incision (Joel–Cohen incision) with being more straight and its modification with fewer usage of sharp instruments, called Misgav–Ladach method (26). These techniques provided shorter operation time and faster recovery with fewer wound infections (27). Nevertheless, in these techniques, the skin incision was several centimeters higher (above the underwear or bikini level) than the original Pfannenstiel incision; moreover, it needs at least 15–17 cm in length (27), which means that these techniques’ abdominal incision looks wider than ours and much higher in level than the original Pfannenstiel incision. Joel–Cohen and Misgav–Ladach techniques did not meet the esthetic expectations of many women (28); however, Belci et al. showed better long-term postoperative results (5 years after CS) in terms of the neuropathic and chronic pain and the cosmetic appearance of the scar in a comparative study (29).

A modified version of the Misgav–Ladach method, which uses the same principles as the original technique but makes the abdominal incision at the same level as the Pfannenstiel incision, gained popularity among obstetricians (28, 30). However, contrary to our modification of the Pfannenstiel incision, all these above techniques focused on the incision level or the surgical techniques rather than the incision length.

## Strength and limitations

The current study is the first to investigate the Pfannenstiel incision length estimation according to the ultrasonographic measurement of the OFD of the fetal head and define a novel technique. The number of cases and design are the main limitations of the study. Only three surgeons used this novel technique. The length of the incision was the primary outcome; therefore, esthetic evaluation was not performed among the women. Also, long-term maternal outcomes of the current technique were not evaluated in this study.

## Conclusion

According to our study findings, the Pfannenstiel incision can be adjusted according to the OFD with minimal margins of error. This modification allows the Pfannenstiel incision length to be kept as fetal head OFD length and may avoid unnecessarily wide incisions providing better cosmetic results without newborn issues or problems in selected patients. Further studies are needed to understand this technique better.

## Data Availability

The raw data supporting the conclusions of this article are available upon request to the corresponding author.

## Supplementary material

Supplementary Videos S1, S2 Demonstration of the surgical technique: Pfannenstiel skin incision according to the fetal occipitofrontal diameter during cesarean delivery (*Pfannenstiel skin incision with Kaya modification*).
